# Scabies Surrepticius: Scabies Masquerading as Pityriasis Rosea

**DOI:** 10.7759/cureus.1961

**Published:** 2017-12-19

**Authors:** Katherine M Stiff, Philip R Cohen

**Affiliations:** 1 Student, Northeast Ohio Medical University; 2 Department of Dermatology, University of California, San Diego

**Keywords:** feces, mite, ova, pityriasis, rosea, scabies, scybala, surrepticius

## Abstract

Scabies, a mite infestation caused by 'Sarcoptes scabiei', most commonly presents as pruritic linear burrows where the mite has invaded the skin. Scabies variant such as bullous, crusted, hidden, incognito, nodular and scalp-mimic the other conditions. In addition, atypical presentations of scabies can masquerade as dermatitis herpetiformis, ecchymosis, Langerhans cell histiocytosis, systemic lupus erythematosus, urticaria, and urticaria pigmentosa. A 59-year-old male presented with non-pruritic papulosquamous plaques on his chest, abdomen, and back resembling lesions of pityriasis rosea in morphology and distribution. The complete cutaneous examination also demonstrated burrows on his finger webs. A mineral oil preparation of skin scrapings showed scabies mites, ova, and scybala. His infestation resolved after the treatment with topical permethrin 5% cream and oral ivermectin 15 mg on days one and eight. In conclusion, scabies surrepticius is a term that has recently been established to unify not only the numerous variants but also the atypical presentations of scabies. The inaccurate diagnosis of scabies infestation can lead to increased medical costs and the side effects of unnecessary tests and the treatment. Pityriasis rosea-like scabies can be added to the list of atypical presentations that are included under the unifying designation scabies surrepticius.

## Introduction

Scabies is a parasitic infestation caused by the mite Sarcoptes scabiei* *[1-3]. Pityriasis rosea is a papulosquamous dermatosis that typically affects the chest, abdomen, and back [4]. A male with scabies, confirmed by the evaluation of a mineral oil preparation of the skin scrapings is described in this case, who presented with papulosquamous plaques that had a morphology and distribution resembling pityriasis rosea.

## Case presentation

A 59-year-old African American male with alcohol-related dementia presented with scaly lesions. His asymptomatic rash had progressively worsened during the prior three months. No one else in his group home had a similar skin condition.

**Figure 1:**
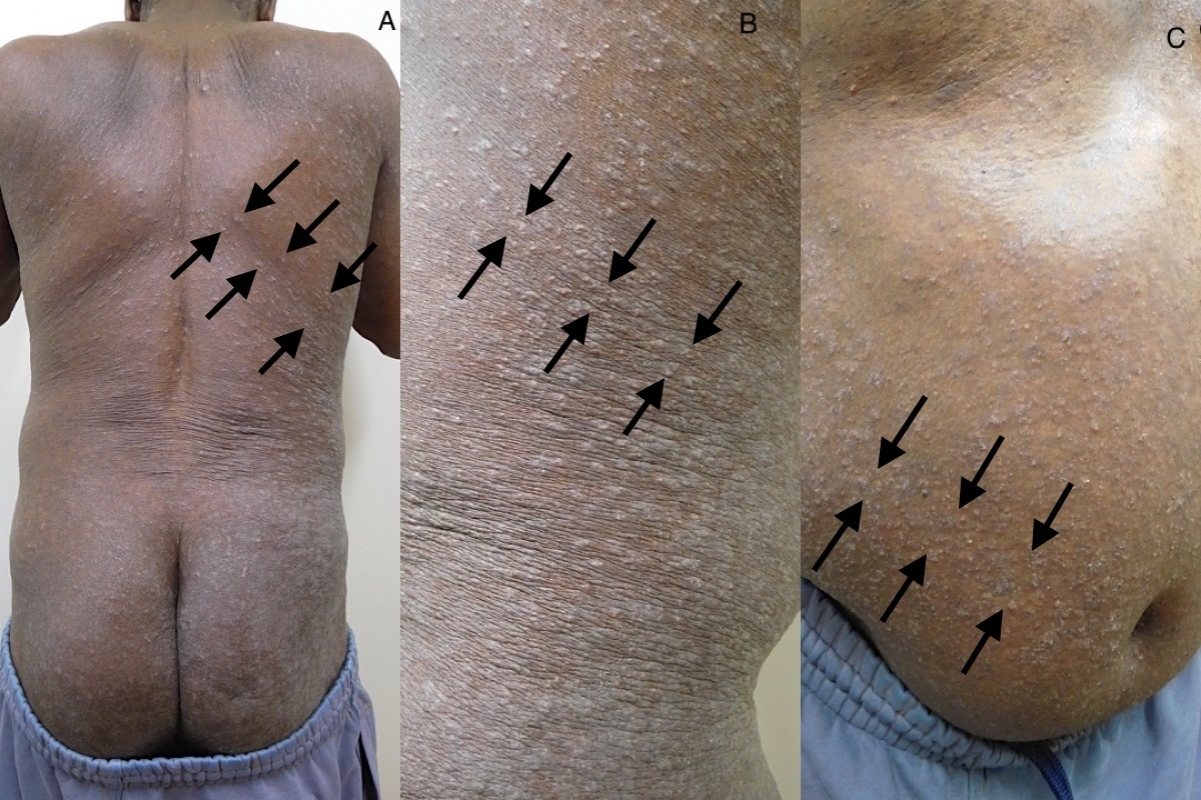
Scabies surrepticius: scabies mimicking pityriasis rosea. Scaly papules and plaques on the back and buttocks (A), the right flank (B), and the right abdomen (C) of a 59-year-old African American male. The skin lesions are distributed along the lines of Langer (indicated by arrows).

**Figure 2:**
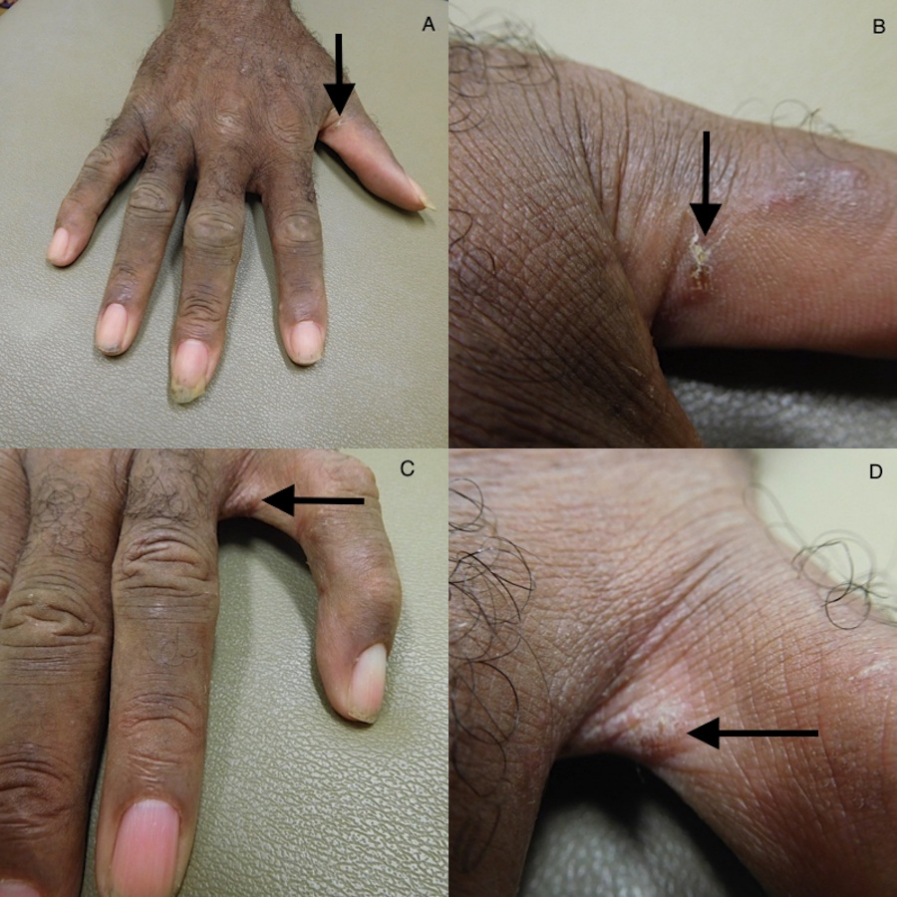
The burrows of the Sarcoptes scabiei mite infestation. The distant (A and C) and closer (B and D) views of the burrows (arrow) on the right thumb and the web spaces between the left fourth and fifth digit.

**Figure 3:**
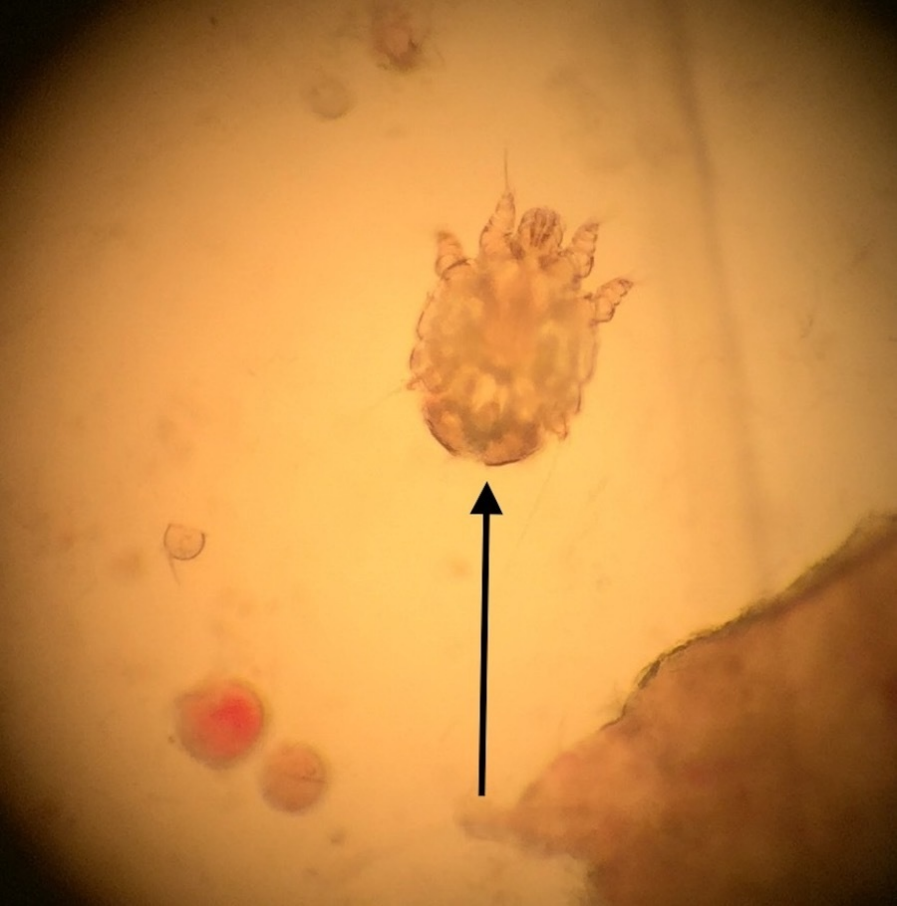
Sarcoptes scabiei mite. The examination of the mineral oil preparation of the skin lesion scrapings using a light microscope shows a Sarcoptes scabiei mite (indicated by the arrow).

The cutaneous examination showed scaly papules and annular plaques on his back, buttocks, flanks, and the abdomen, many of them were distributed along the lines of Langer (Figure 1). Similar lesions were absent on his face, neck, arms, forearms, and legs. However, closer examination revealed burrows on his finger webs (Figure 2). Separate mineral oil preparations of the skin scrapings taken from several areas including his chest, finger webs, and umbilicus, each showed mites, ova, and scybala (Figure 3).

The correlation of the clinical presentation and mineral oil preparations confirmed the diagnosis of scabies masquerading as pityriasis rosea. The patient was treated topically with permethrin 5% cream and also received oral ivermectin 15 mg on days one and eight.

The patient returned for follow-up examination four weeks later. There was complete resolution of the skin lesions. In addition, separate mineral oil skin preparations taken from multiple sites were each negative for mites, ova, and scybala.

## Discussion

Scabies is common and occurs worldwide with an estimated yearly global prevalence of 200 million [5]. Indeed, the World Health Organization declared scabies to be a “neglected skin disease” in 2009 [6]. It is epidemic in the tropical regions, such as Papua New Guinea, Panama, and Fiji [7]. In developing countries, scabies is frequently observed in children; however, in industrial nations, it is more often diagnosed in long-term care facilities, hospitals, and areas of over-crowding [1].

The mite Sarcoptes scabiei completes its life cycle in humans. The transmission is most common through skin-to-skin contact, but can also occur through infested bedding or clothing. The mite burrows into the skin, where the female lays her eggs. The clinical presentation of scabies is due to a combination of the mite infestation and a hypersensitivity reaction to the mite and its excrement [1].

Scabies is suspected based on a history of pruritic lesions, most commonly located in the finger webs or flexor surfaces of the elbows and wrists. The lesions have also been found in the areola, axillae, belt line, buttocks, genitalia, and umbilicus [1-3]. The itching is usually worse at night. Also, it is common for household members to have similar symptoms.

There are numerous clinical variants of scabies, including bullous, crusted, hidden, incognito, nodular and scalp. Scabies has also been found to mimic dermatitis herpetiformis, ecchymosis, Langerhans cell histiocytosis, systemic lupus erythematosus, urticaria, and urticaria pigmentosa [3]. Scabies surrepticius has been proposed as a unifying term to describe the atypical morphological presentations of this infestation (Table 1) [3].

**Table 1:** Table representing the subtypes of the scabies surrepticius.

Scabies surrepticius subtypes
Bullous
Crusted
Dermatitis herpetiformis-like
Ecchymoses
Hidden
Incognito
Langerhans cell histiocytosis-like
Nodular
Pityriasis rosea-like
Scalp
Systemic lupus erythematosus-like
Urticaria
Urticaria pigmentosa-like

Scabies is diagnosed by demonstration of the mites, eggs, or scybala using a light microscope to evaluate the skin scrapings from the lesions [1-3]. The dermoscopy can also be used to diagnose scabies. The mites appear as a triangular structure with a trailing burrow when viewed with the dermatoscope [8].

The diagnosis of scabies can be challenging due to its ability to masquerade as other conditions. A recent study found that 45% of the patients presenting to the dermatology office with scabies had been misdiagnosed by another provider [9]. The misdiagnosed patients underwent unnecessary costs for the tests and treatment. Increasing awareness of the atypical presentations of scabies can reduce the financial burden and the possible side effects of the treatment due to an incorrect diagnosis.

The initial clinical impression of pityriasis rosea during the preliminary evaluation of our patient was prompted by not only the absence of pruritus, but also the morphology and distribution of the skin lesions on his trunk; there were numerous scaly oval plaques along Langer's lines of cleavage in a fir-tree distribution [4]. However, the closer examination led us to perform skin scrapings for a mineral oil preparation based upon the identification of the burrows on his finger webs. We speculate that the absence of pruritus in our patient may be due to his alcohol-related dementia, decreased peripheral sensation secondary to chronic alcohol use, or both.

## Conclusions

Scabies classically presents with the pruritic burrows in the finger webs. However, numerous atypical presentations of scabies, referred to as scabies surrepticius, have been described. A male with scabies whose skin lesions masqueraded as pityriasis rosea is described in this case. Therefore, scabies should be included in the differential of pityriasis rosea and pityriasis rosea can be added as a subtype of scabies surrepticius.
